# ENIGMA brain injury: Framework, challenges, and opportunities

**DOI:** 10.1002/hbm.25046

**Published:** 2020-06-01

**Authors:** Emily L. Dennis, David Baron, Brenda Bartnik‐Olson, Karen Caeyenberghs, Carrie Esopenko, Frank G. Hillary, Kimbra Kenney, Inga K. Koerte, Alexander P. Lin, Andrew R. Mayer, Stefania Mondello, Alexander Olsen, Paul M. Thompson, David F. Tate, Elisabeth A. Wilde

**Affiliations:** ^1^ Department of Neurology University of Utah School of Medicine Salt Lake City Utah USA; ^2^ George E. Wahlen Veterans Affairs Medical Center Salt Lake City Utah USA; ^3^ Imaging Genetics Center Stevens Neuroimaging & Informatics Institute, Keck School of Medicine of USC Marina del Rey California USA; ^4^ Western University of Health Sciences Pomona California USA; ^5^ Department of Radiology Loma Linda University Medical Center Loma Linda California USA; ^6^ Cognitive Neuroscience Unit, School of Psychology Deakin University Burwood Victoria Australia; ^7^ Department of Rehabilitation and Movement Sciences Rutgers Biomedical Health Sciences Newark New Jersey USA; ^8^ Department of Psychology Pennsylvania State University University Park Pennsylvania USA; ^9^ Social Life and Engineering Sciences Imaging Center University Park Pennsylvania USA; ^10^ Department of Neurology Uniformed Services University of the Health Sciences Bethesda Maryland USA; ^11^ National Intrepid Center of Excellence Walter Reed National Military Medical Center Bethesda Maryland USA; ^12^ Psychiatry Neuroimaging Laboratory Brigham and Women's Hospital Boston Massachusetts USA; ^13^ Department of Child and Adolescent Psychiatry, Psychosomatics and Psychotherapy Ludwig‐Maximilians‐Universität Munich Germany; ^14^ Center for Clinical Spectroscopy Brigham and Women's Hospital, Harvard Medical School Boston Massachusetts USA; ^15^ Mind Research Network Albuquerque New Mexico USA; ^16^ Department of Neurology and Psychiatry University of New Mexico School of Medicine Albuquerque New Mexico USA; ^17^ Department of Biomedical and Dental Sciences and Morphofunctional Imaging University of Messina Messina Italy; ^18^ Department of Psychology Norwegian University of Science and Technology Trondheim Norway; ^19^ Department of Physical Medicine and Rehabilitation St. Olavs Hospital, Trondheim University Hospital Trondheim Norway; ^20^ Department of Neurology, Pediatrics, Psychiatry, Radiology, Engineering, and Ophthalmology University of Southern California (USC) Los Angeles California USA

**Keywords:** brain injury, concussion, ENIGMA, neuroimaging, TBI

## Abstract

Traumatic brain injury (TBI) is a major cause of disability worldwide, but the heterogeneous nature of TBI with respect to injury severity and health comorbidities make patient outcome difficult to predict. Injury severity accounts for only some of this variance, and a wide range of preinjury, injury‐related, and postinjury factors may influence outcome, such as sex, socioeconomic status, injury mechanism, and social support. Neuroimaging research in this area has generally been limited by insufficient sample sizes. Additionally, development of reliable biomarkers of mild TBI or repeated subconcussive impacts has been slow, likely due, in part, to subtle effects of injury and the aforementioned variability. The ENIGMA Consortium has established a framework for global collaboration that has resulted in the largest‐ever neuroimaging studies of multiple psychiatric and neurological disorders. Here we describe the organization, recent progress, and future goals of the Brain Injury working group.

## Graphical abstract

Graphical Abstract

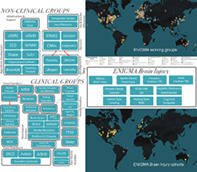

## 1 INTRODUCTION

The ENIGMA Brain Injury working group was formed in 2016 with the overall aim of finding reproducible neuroimaging biomarkers of traumatic brain injury (TBI) across the injury spectrum. TBI is defined as an alteration in brain function, or other evidence of brain pathology, caused by an external force (Menon et al., 2010). Injury severity ranges from devastating brain damage causing death or severe disability to milder injuries, including concussions and subconcussive impacts. TBI is a major public health issue around the world, with an estimated 70 million people sustaining a TBI each year (Dewan et al., 2018). While some patients recover after TBI, others experience chronic physical, cognitive, and psychological dysfunction. Long‐term disability is more common in moderate and severe TBI (generally injuries with a Glasgow Coma Scale [GCS] < 12), but a considerable proportion of persons with mild TBI experience persistent health problems. There is a considerable amount of variance in outcome that is not explained by typical injury‐related factors. It is likely that patient subgroups, even within defined patient populations (e.g., pediatric moderate/severe TBI patients), have different trajectories of recovery or different constellations of symptom burden.

**FIGURE 1:**
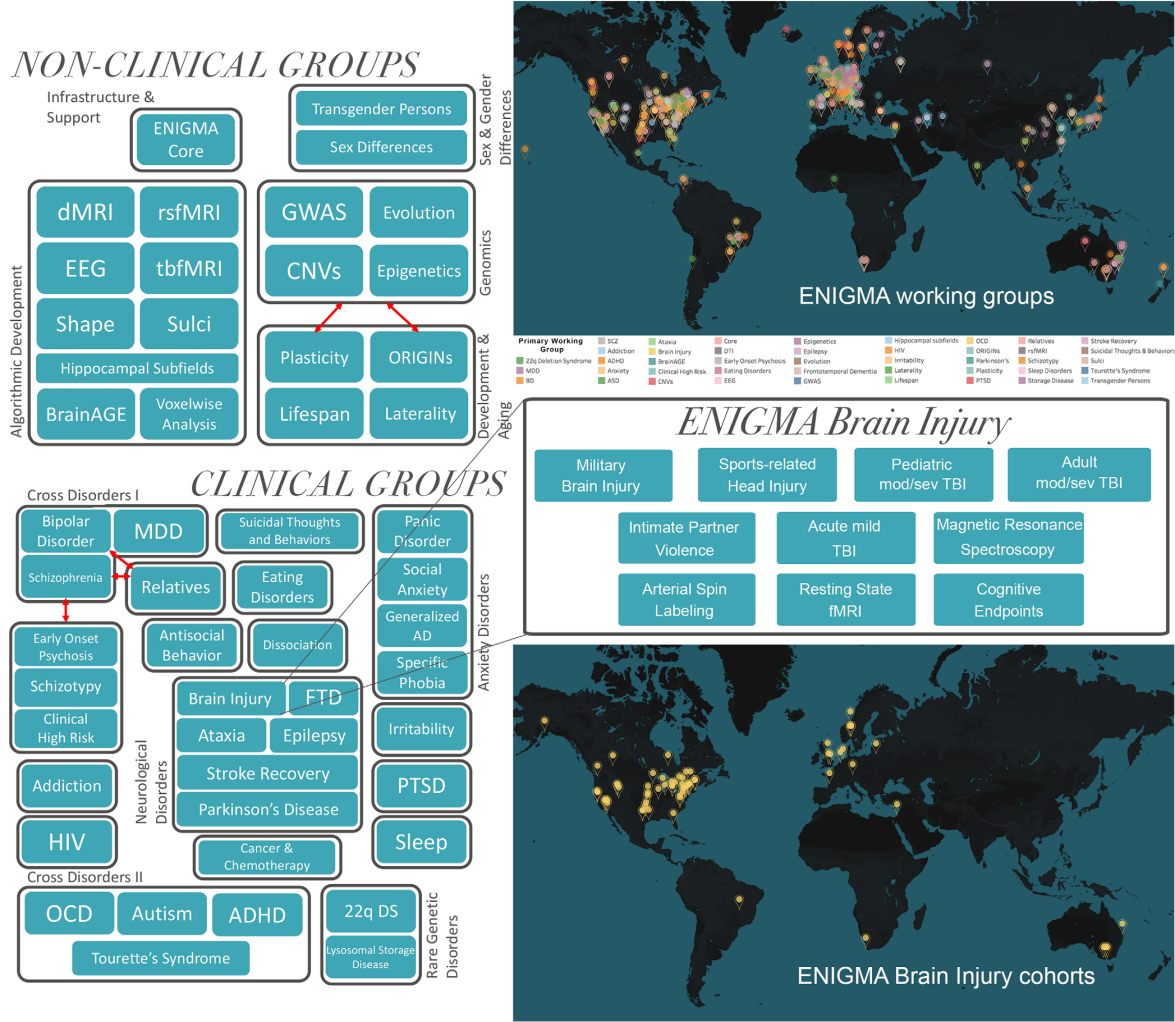
Organization of ENIGMA and the Brain Injury working group. 22q DS, 22q11.2 deletion syndrome; AD, anxiety disorder; ADHD, attention‐deficit/hyperactivity disorder; CNV, copy number variation; dMRI, diffusion magnetic resonance spectroscopy; EEG, electroencephalography; FTD, frontotemporal dementia; GWAS, genome‐wide association study; HIV, human immunodeficiency virus; MDD, major depressive disorder; OCD, obsessive compulsive disorder; PTSD, posttraumatic stress disorder; rsfMRI, resting state functional MRI; tbfMRI, task‐based fMRI; TBI, traumatic brain injury. Adapted from Thompson et al., 2020

With large sample sizes and big data approaches, the ENIGMA Brain Injury working groupaims to shed light on this possibility. Within the ENIGMA Brain Injury working group, there are now six subgroups based on patient population and injury mechanism, and four subgroups focused on methods development. The clinical groups are Pediatric Moderate/Severe TBI (msTBI), Adult msTBI, Military Brain Injury, Sports‐Related Head Injury, Intimate Partner Violence (IPV), and Acute Mild TBI. The methods groups are Magnetic Resonance Spectroscopy, Arterial Spin Labeling, Resting‐State fMRI, and Cognitive Endpoints (Figure 1). The methods groups are focusing on building standardized and novel methods of analysis and developing approaches for post hoc harmonization of data that has already been collected with varying parameters at different sites. However, to overcome the challenges of TBI complexity and heterogeneity and make progress in clinical classification while improving the diagnostic and management process, a multimodal strategy is crucial. Thus, across each of these groups, we plan to evaluate, when possible, fluid and genetic biomarkers, in addition to neuroimaging analyses, to better unveil the pathobiological basis of TBI. A number of large TBI research initiatives, including TRACK‐TBI, CENTER‐TBI, and CENC‐LIMBIC, have measured fluid biomarkers (Maas et al., 2015; Walker et al., 2016; Yue et al., 2019). The ENIGMA Brain Injury working group will bank on these initiatives and leverage their knowledge and legacy data to identify novel ways in which fluid and imaging modalities can be combined to refine patient characterization, deliver precision medicine and, ultimately, improve outcomes after TBI.

For more details on the working groups including more depth regarding population‐specific considerations (i.e., blast TBI in military or repetitive impacts in sports) and planned analyses, we refer the interested reader to a recent special issue to be published in *Brain Imaging and Behavior* (preprints currently available on PsyArxiv; Bartnik‐Olson et al., in press; Dennis et al., in press; Esopenko et al., 2019; Koerte et al., 2020; Olsen, et al., 2020; Tate et al., 2019; Wilde, Dennis, & Tate, 2019). Across these working groups, there are currently over 150 collaborators involved from 76 institutions and 13 countries.

In this article, we will discuss some of the challenges and open questions in the field of TBI neuroimaging research, including challenges specific to the meta‐ and mega‐analytical approaches of the ENIGMA Consortium. A wide range of factors can impact outcomes after brain injury, and this contributes to the large heterogeneity in outcome. Broadly speaking, heterogeneity is one of the largest challenges in TBI research as it complicates diagnosis and prognosis. However, this is also an opportunity, as we can reasonably assume that this heterogeneity is not random and likely indicates that there are individual factors or combinations of factors that play a role in outcome, perhaps in ways that are too subtle to have been detected yet. The ENIGMA Brain Injury group aims to uncover some of these factors, to improve the amount of variability in outcomes we are able to explain. Some of the challenges in TBI research discussed below include choice of comparison groups, consideration of lesions or other factors that may introduce significant distortion in various MRI sequences, inconsistently applied diagnostic criteria (particularly for mild TBI), and complexity and multiplicity of the underlying pathology. In addition to these general challenges in TBI research, there are a number of challenges in the ENIGMA approach. Data harmonization is a significant challenge and must be addressed carefully. If this is not done in a thoughtful way, we risk a “garbage in, garbage out” scenario that yields null results at best, or unreliable or inaccurate results at worst. Harmonization includes neuroimaging, demographic, clinical, and cognitive/neuropsychological data. These challenges and our approach to addressing them in the specific context of TBI are discussed further in the *Challenges and Opportunities* section below.

### 1.1 ENIGMA as a vehicle for reproducible science

There is no corner of modern science left untouched by the rippling effects of the “reproducibility crisis” and, in some cases, well‐celebrated findings have been challenged or overturned (Oostenbroek et al., 2016; Simmons & Simonsohn, 2017). While this crisis has been building for some time (Gelman, 2016; Ioannidis, 2005; Meehl, 1967), a clear watershed moment in the social sciences was the 2015 paper published by Nosek and colleagues which made clear that successful replication of findings is indeed quite challenging, hovering at ~36% (Open Science Collaboration, 2015). The scientific discourse since the work by Open Science Consortium (OSC) has focused, to some extent, on identifying mechanisms for our scientific failures leading to the inevitable critiques about how science is conducted. Concerns have ranged from the widespread reliance upon null hypothesis significance testing (Benjamin et al., 2018; Schneider, 2015), to the failure to adequately control false positives (Smith & Nichols, 2018), to the incentive structure that does not emphasize, or worse, may even penalize, open and transparent science (Gernsbacher, 2018), to the flexibility in approach or “investigator degrees of freedom” (Bowring, Maumet, & Nichols, 2019; Hallquist & Hillary, 2019; Simmons, Nelson, & Simonsohn, 2011) and funding agencies focused on research programs of innovation over careful work establishing ground truths, including replication (Lilienfeld, 2017).

While the crisis provided an initial jolt to the research community—including some challenges to the goals of “open science” (Mirowski, 2018) and the narrative framed by the reproducibility crisis (Fanelli, 2018)—with growing acceptance that science is, of course, imperfect, several downstream effects of this crisis have been positive. In the neuroimaging community, the crisis has evoked a period of scientific introspection, with investigators revisiting their methods and fundamental assumptions about how they engage in their science (Hillary & Medaglia, in press; Nichols et al., 2017; Poldrack et al., 2017). Investigators may have also become more open to the prospect of sharing their data, and the neuroimaging community is actively organizing itself to develop methods to meet this opportunity, which is itself a nontrivial endeavor (Poldrack & Gorgolewski, 2014). The ENIGMA Brain Injury effort now operates at this seemingly pivotal moment in the modern scientific timeline, where scientific communities are now leveraging shared data and ideas to (re)establish ground truths, representing the foundation of our science. In doing so, efforts through ENIGMA for data sharing, and open science, could represent a paradigm shift in not only how we engage our science also how we engage one another (Hillary & Medaglia, in press; Poldrack et al., 2017). The ENIGMA Brain Injury initiative is based on the principles of transparency, rigor, reproducibility, and collaboration, and is well‐aligned with goals for reproducible science, by integrating scientists and increasing sample sizes to garner robust and reliable findings.

## 2 PRIMARY AIMS

The overall aims of the ENIGMA Brain Injury group are as follows.

1. Establish a collaborative international network of researchers focused on TBI, including neuroimaging, clinical, cognitive, neuropsychological, and fluid biomarker data
2. Develop and test new pipelines for processing neuroimaging data that include consideration of lesions and allow for longitudinal modeling of injury/recovery
3. Support the advancement of junior investigators through opportunities to lead analyses
4. Provide a forum for researchers to discuss and debate unresolved scientific questions

Some of the research questions that apply broadly across the sub‐working groups include:

1. Sex differences in postinjury outcome
2. The influence of comorbid disorders (such as PTSD or ADHD) on outcome
3. Identification of clinically‐meaningful patient subgroups

In addition, each working group has research questions specific to their patient population which are discussed in greater detail in recent papers by each subgroup (Dennis, et al., in press; Esopenko et al., 2019; Koerte et al., 2020; Olsen, et al., 2020; Tate et al., 2019).

## 3 CHALLENGES AND OPPORTUNITIES

### 3.1 Sex differences

In the general population, males sustain TBI at a higher rate than females (Langlois, Rutland‐Brown, & Wald, 2006). This may be for a variety of reasons, including engaging in activities with a risk of TBI at a greater frequency (such as military service or risky physical activity) or due to sex differences in premorbid conditions that may increase vulnerability for TBI (such as attention‐deficit/hyperactivity disorder). However, this difference is only present between puberty and middle age. In young children and older adults, the rates of TBI in males and females are approximately equal (Langlois et al., 2006). Some early studies have shown worse outcome in females across the severity spectrum (Farace & Alves, 2000), but this remains an active area of investigation. One particular patient population that is understudied includes individuals sustaining head trauma as a result of intimate partner violence (IPV). Although women tend to experience more severe physical trauma than men resulting in a high risk of head injury in IPV, the ENIGMA IPV working group will examine the effects of IPV‐related TBI, including the often repetitive nature of the injuries and the multitude of comorbid disorders and polytrauma that may result, in both men and women (Esopenko et al., 2019). Sex differences have become a major topic in TBI and concussion research in recent years, and the ENIGMA Brain Injury working group will contribute to this body of knowledge.

With regard to mild TBI and especially sports‐related head injury, the rate of injury in females may be higher than previously assumed, as research has focused on traditionally male‐dominated sports like American football. Females of all ages are at an increased risk of sustaining a sports‐related concussion (Abrahams, Fie, Patricios, Posthumus, & September, 2014). Female participation in the National Collegiate Athletic Association is at an all‐time high, and an estimated 43% (~210,000) of all collegiate student‐athletes are girls and women (NCAA, 2017). Nonetheless, despite the high number of female athletes, females remain an understudied population in sports‐related concussion (O'Reilly, Wilson, & Peters, 2018). There may be structural brain differences that influence risk for concussion, as a recent study found smaller diameter axons in females, which was associated with increased vulnerability to shear deformation (Dollé et al., 2018). Although females have an increased risk of sustaining a concussion compared to males, and although there is evidence for increased vulnerability to shear deformation, only a small number of studies systematically include both female and male individuals to investigate sex differences following a concussion. The available studies suggest that females report more symptoms and greater symptom severity acutely as well as 3 months postinjury (Frommer et al., 2011; Merritt & Arnett, 2014; Mihalik et al., 2013; Ono et al., 2016; Preiss‐Farzanegan, Chapman, Wong, Wu, & Bazarian, 2009). Moreover, females tend to take longer to recover and have an increased risk for worse neurological (i.e., worse vestibular–oculomotor function, increased risk of posttraumatic migraine) (Henry, Elbin, Collins, Marchetti, & Kontos, 2016; Howell, Stracciolini, Geminiani, & Meehan 3rd, 2017; Mihalik et al., 2013; Sufrinko et al., 2017) and cognitive (i.e., worse visual memory, longer reaction time; Broshek et al., 2005; Chiang Colvin et al., 2009; Covassin, Elbin, Bleecker, Lipchik, & Kontos, 2013; Covassin, Elbin, Harris, Parker, & Kontos, 2012; Covassin, Schatz, & Swanik, 2007; Léveillé, Guay, Blais, Scherzer, & De Beaumont, 2017; Sandel, Schatz, Goldberg, & Lazar, 2017; Sicard, Moore, & Ellemberg, 2018) outcomes following concussion compared to males. While studies of sex differences in neurobehavioral outcome after concussion are limited, data so far suggest that rates of depressive symptoms after injury are similar between males and females (Kontos, Covassin, Elbin, & Parker, 2012). However, future studies using more comprehensive neuropsychological batteries are needed to objectively assess sex differences post‐SRC.

State‐of‐the‐art neuroimaging techniques such as diffusion MRI (dMRI) and magnetic resonance spectroscopy (MRS) may provide objective measures of sex‐specific differences (Panchal et al., 2018; Sollmann et al., 2018). However, there are currently few articles on sex differences following concussion using these potentially useful neuroimaging techniques (Chamard et al., 2012; Fakhran, Yaeger, Collins, & Alhilali, 2014; Helmer et al., 2014; Hsu et al., 2016). These existing studies collectively reveal differences in imaging measures (e.g., white matter diffusion, microhemorrhages, *N*‐acetylaspartate [NAA]) following concussion. Yet, it remains unclear to what extent the reported disparity in neuroimaging outcomes between males and females post‐SRC is due to potential premorbid differences (e.g., axonal structure; Dollé et al., 2018). Nonetheless, neuroimaging techniques can be harnessed to provide quantitative measures that provide insight into sex‐specific differences in underlying pathological pathways of injury. The ENIGMA approach aims to combine data from various studies to understand the clinical implications of sex‐specific differences following concussion.

### 3.2 Chronic effects of TBI and risk for neurodegeneration

Convergent evidence in animal and epidemiological work points to TBI as a risk factor for neurodegeneration later in life (Dams‐O'Connor et al., 2013; Itoh et al., 2009; McKee et al., 2009; Tran, LaFerla, Holtzman, & Brody, 2011; Williams, Peltz, Yaffe, Schulz, & Sierks, 2018). Yet much less work examines the very long‐term consequences of TBI (in particular, a single moderate‐to‐severe event) as individuals progress into older adulthood. Prospective investigation of the consequences of TBI is vital given that normal aging amplifies the effects of TBI over time (Moretti et al., 2012) and recent work has also linked msTBI to other pathology including tauopathy and synucleinopathy (Acosta et al., 2015; Dams‐O'Connor et al., 2013; Tan et al., 2019; Williams et al., 2018). There remains little information on how TBI interacts with distinct pathways to neurodegeneration including impaired immune function/inflammation (Heneka et al., 2015), vascular risk and associated white matter degradation (Sweeney, Kisler, Montagne, Toga, & Zlokovic, 2018; Zlokovic, 2011), and alterations in large‐scale neural networks (Hillary & Grafman, 2017; Jones et al., 2016), leaving the precise relationship between TBI and risk for neurodegeneration unknown. Addressing this issue has become a primary health care concern in the study of chronic TBI. One important contributor to the lack of prospective work in older chronic TBI (e.g., >10 years postinjury, >50 years of age) is that recruitment of these clinical samples is challenging for any single site, resulting in either heterogeneous samples—with respect to age and time postinjury—or smaller samples underpowered to examine multivariate effects. To understand the complex interaction of factors that give rise to the behavioral phenotypes reflected in the population of individuals aging‐with‐TBI, multi‐site efforts will be vital. With increased access to data and integration of multiple sources of expertise, the ENIGMA initiative offers unique advantages in efforts to harmonize imaging, blood biomarkers, and genetic data to isolate distinct outcome trajectories in chronic TBI.

### 3.3 Comparison groups

The issue of what constitutes an optimal control group is an open question across the field of TBI research. In military brain injury, for example, deployed (but uninjured) Service Members represent a common comparison group, but potential comparisons within injured Service Members are many: Blast‐related versus impact‐only injury, blast‐related TBI versus blast‐exposed without TBI, combat‐related TBI versus noncombat deployment‐related TBI, single versus few versus many exposures to potential concussive events, among others. In pediatric TBI, studies typically include either healthy children (HC) or children with orthopedic injury (OI) as controls, or compare moderate and severe TBI to mild TBI (Wilde et al., 2019). Some premorbid conditions may increase vulnerability to injury, such as ADHD, and postinjury factors such as medication and extended medical treatment may motivate the choice of OI over HC (Hajek et al., 2010; Holbrook et al., 2005). Even so, there may also be preinjury and postinjury differences between TBI and OI (Basson et al., 1991; Borsook, 2012; Loder, Warschausky, Schwartz, Hensinger, & Greenfield, 1995; Wilde, Ware, et al., 2019). Considerations for selection of comparison groups are similar to adult msTBI. Sample sizes to date have generally been too limited to fully address this question, but ENIGMA Brain Injury has the opportunity to bring additional light to this area. In sports‐related head injury, the choice of comparison group depends on the clinical population being studied (Gardner et al., 2012; Koerte et al., 2015; Manley et al., 2017). When examining the effects of concussion, uninjured athletes, either from contact or noncontact sports may serve as controls, and this is generally preferable to using nonathletes as controls due to the presumed unique characteristics associated with the habits, physiology, and fitness level of some athlete groups in addition to their relative risk of exposure to repetitive head hits (RHH) (Koerte, Hufschmidt, Muehlmann, Lin, & Shenton, 2016; McCrea et al., 2017). When examining the impact of RHH, noncontact athletes or nonathletes may be studied, or preseason and postseason assessments allow for the participant to serve as their own control (Koerte et al., 2016; Koerte, Lin, Willems, et al., 2015; Lin et al., 2015). For the study of IPV‐related brain injury, comparison to healthy controls is likely not appropriate for some questions as studies cannot account for chronic exposure to multiple forms of trauma including psychological, sexual, and physical abuse as an outcome of IPV, as well as the repetitive nature of brain trauma in this population. Furthermore, regardless of head trauma history, those who have experienced IPV often present with a number of comorbid psychopathologies such as PTSD, depression, anxiety, and substance use disorders. Thus, comparison to other individuals with exposure to IPV, but without exposure to brain injury, is likely required in these studies. The ENIGMA platform provides an opportunity for researchers in this field to examine the impact of comparison groups and to better understand the differences between these populations. Linked with the question of optimal comparison groups is the issue of comorbidities, as mentioned above. As discussed further in the *Future Directions* section, there are numerous existing psychiatric working groups within ENIGMA, enabling well‐powered, cross‐disorder analyses. One piece that is critical in the identification of appropriate comparison groups is a full accounting of lifetime TBI exposure. There are a number of scales available for assessing lifetime exposure, some more general and others more tailored to specific patient populations (e.g., Ohio State University TBI Identification method [OSU‐TBI‐ID] or the Boston Assessment of TBI—Lifetime [BAT‐L]). We recommend that groups use an appropriate scale for assessing lifetime TBI exposure, but lack of complete data in this area is inevitable in some legacy datasets.

### 3.4 Combining nonequivalent MRI data

With the goal of advancing multi‐site MRI data collection and sharing, there has been significant effort over the past two decades to facilitate data collection that is comparable in terms of acquisition parameters, sequences employed, and scanner environment. Concerns about these classic issues for data sharing have resulted in vital protocols developed to optimize reliable and high‐value MR data acquisition across data collection sites (see BIRN; Friedman et al., 2006; Friedman et al., 2008). The goal for ENIGMA to combine archival (dark) data sets (Hawkins, Huie, Almeida, Chen, & Ferguson, 2019) holds unique challenges for data harmonization beyond those posed by traditional methods for MRI parameterization (e.g., repetition time, slices, volumes). Beyond the clear examples where physical parameters are known to influence MRI data quality (e.g., signal‐to‐noise ratio is positively correlated with magnetic field strength), what is less clear is the inequivalent data acquisition that would render data incomparable. There is enormous potential for combining archival data in MRI; however, the challenges in combining data are just now beginning to be understood with novel methods emerging with the motivation to harmonize nonequivalent data (Adhikari et al., 2018; Badhwar et al., 2020; Jahanshad et al., 2013, 2019; Potvin et al., 2019; Saemann et al., 2019). There is emerging support for several methods for combining MRI data including linear scaling functions like ComBat (Johnson, Li, & Rabinovic, 2007) and more recent adaptations of ComBat that include nonlinear scaling (e.g., ComBat‐GAM) to examine volumetric changes over the lifespan of healthy adults (Pomponio et al., 2020). These developments are encouraging, but issues persist when extending these methods to examine scanner and time effects on functional datasets, and harmonization approaches, and data processing pipelines are not standardized (e.g., for cortical thickness, 3D lesion mapping, static and dynamic functional connectivity). To leverage the advantage of archival data, our working group will test and develop methods that allow investigators to combine nonequivalent data where data parameters differ, for example, the spatial or angular resolution of anatomical and diffusion MRI, or the specific protocols for collecting structural MRI, EEG, rsFMRI, or MRS data. While combining this type of data has historically been viewed as taboo or even impossible (with respect to data fidelity), there are recent efforts to examine these assumptions, including the critical breaking‐points where MRI data can and cannot be reliably combined. As part of this goal, examiners aim to identify the types of variables that are the most robust to combining high quality, nonequivalent data. For example, through data collection across a range of parameters (TR, TE, slices, volumes, amounts of participant motion), examiners can probe where differences in acquisition have the greatest influence on measures such as brain volumetrics, diffusion measurements, and even structural and functional connectivity modeling, with the goal of examining how variation in data acquisition may influence both group differences and their relation to clinical data and outcome. Ultimately, advancing data harmonization is fundamental to ENIGMA efforts, and success in our and other working groups will serve the imaging community broadly.

### 3.5 Lesion identification and modeling for image registration

Given the nature of TBI, where an external physical force has disrupted the central nervous system, one common result is a gross abnormality of brain tissue (i.e., brain lesions). While there is a long history of using brain lesions as a predictor of outcome in TBI (Levin et al., 1988), for the goals of the ENIGMA Brain Injury working group, lesions pose methodological problems for image registration (Wang et al., 2012) and may even alter the signal in functional MRI (Hillary & Biswal, 2007; Lee, Polimeni, Price, Edlow, & McNab, 2018). Therefore, one critical goal for the ENIGMA Brain Injury working group is to develop and test methods that aid in image processing for characterization, mapping, and quantification of lesions (Wang et al., 2014) across different MRI sequences, and also modeling of the effects of lesions in brain networks (Irimia, Goh, Torgerson, Vespa, & Van Horn, 2014; Irimia & Van Horn, 2014; Lee et al., 2018; Roy et al., 2017). As an early goal, we are focused on developing distinct lesion mapping decision trees that accommodate the data types available (e.g., T1‐weighted, FLAIR). Moreover, we aim to examine the influence of brain lesions on structural and functional networks directly by first modeling lesions in 3D representation (Irimia et al., 2014) to integrate lesions into networks to understand their direct effects on connectivity dynamics (as an example see (Lee et al., 2018).

### 3.6 Multimodal characterization of injury

While traditional CT is important for initial clinical decision‐making, particularly in the acute and subacute phase following TBI, MR modalities are often preferred in neuroimaging research given their higher sensitivity to some forms of TBI‐related pathology (e.g., diffusion MRI for traumatic axonal injury, susceptibility‐weighted imaging for microhemorrhages, etc.). The use of dMRI in TBI has dramatically expanded recently (Hulkower, Poliak, Rosenbaum, Zimmerman, & Lipton, 2013) and future studies will hopefully incorporate more advanced multi‐shell dMRI, allowing for more detailed information on pathology by modeling diffusion in different cellular compartments (Zhang, Schneider, Wheeler‐Kingshott, & Alexander, 2012). There have been numerous reviews recently on neuroimaging in TBI, so for more information, we refer readers to these (Dennis, Babikian, Giza, Thompson, & Asarnow, 2017, 2018; Koerte, Lin, Willems, et al., 2015; Shenton et al., 2012; Wilde et al., 2015). Here we discuss three approaches—advanced functional MRI analysis, MRS, and structure–function coupling. We acknowledge that there are several other promising forms of structural and functional neuroimaging for use in TBI and concussion, including arterial spin labeling and other forms of perfusion imaging, imaging to measure cerebrovascular response, magnetoencephalography, but comprehensive review of all modalities is beyond the scope of this article.

#### 3.6.1 Advanced fMRI

The blood‐oxygen level‐dependent (BOLD) response represents a complex physiological response through which investigators can examine for both acute and chronic effects of TBI, as well as exposure to repetitive head injury (Eierud et al., 2014; Manley et al., 2017; Mayer, Bellgowan, & Hanlon, 2015; McDonald, Saykin, & McAllister, 2012; Olsen et al., 2015; Talavage et al., 2014). All BOLD methodologies (task‐evoked and resting‐state) have relatively high spatial resolution (2–4 mm voxel size) and the ability to query deep gray matter structures that have been frequently associated with TBI due to both the morphology of the skull and the aggregation of shear stressors in these regions (Bigler & Maxwell, 2012; Zhang, Yang, & King, 2004). In addition, task‐evoked BOLD can be used to directly interrogate the neural basis of cognitive and emotional dysfunction frequently observed posttrauma in many patients. In terms of analyses, the hemodynamic response contains rich information about various neurovascular coupling properties, including inhibitory activity (Mayer et al., 2019). However, to date, only a few studies have explicitly examined these various hemodynamic properties in mild (Mayer et al., 2014) or more severe TBI (Palmer et al., 2010), instead of relying on more traditional analyses that assume a canonical shape of the hemodynamic response function. Others have used evoked BOLD techniques in conjunction with gas challenges to query cerebral vascular reactivity as an additional measure of trauma‐induced pathology (Ellis et al., 2016). More recently, TBI‐related changes in BOLD activation has also been linked to an electrophysiological measure of interhemispheric transfer time of neuronal information (Olsen et al., 2020). Despite potential promise as a marker of successful rehabilitation and target engagement, only a few studies have utilized the BOLD response during pharmacological challenges or following cognitive rehabilitation (Galetto & Sacco, 2017; McAllister et al., 2011).

#### 3.6.2 Magnetic resonance spectroscopy

MRS is a noninvasive method to measure brain metabolites, where each peak or resonance is proportional to the concentration of that metabolite in a specified volume of interest. The exquisite sensitivity of MRS to noninvasively measure key neurometabolites provides a unique window into the brain's response to injury or disease, and combining MRS in multimodal imaging studies can also be valuable as each modality provides information that can be complementary. Since abnormal metabolism is part of the pathophysiologic cascade following traumatic brain injury (TBI), MRS has been utilized to identify metabolite changes in regions of injury, to define the extent of pathology, and as a biomarker of outcome.

Given the heterogeneity of injury and variability in acquisition methods and sampled locations, MRS findings across studies are not always consistent. Nevertheless, the consensus is that *N*‐acetylaspartate (NAA), a marker of neuronal loss or dysfunction, declines, both in acute and postacute patients, with a secondary finding of increased choline, a marker of membrane synthesis or repair, inflammation, or demyelination (Gardner, Iverson, & Stanwell, 2014; Hillary et al., 2007; Kirov, Whitlow, & Zamora, 2018; Lin et al., 2012). These abnormalities are magnified by injury severity as assessed by the Glasgow Coma Scale (GCS), posttraumatic amnesia (PTA), and MRI findings (Garnett, Blamire, Rajagopalan, Styles, & Cadoux‐Hudson, 2000; Govind et al., 2010). Moreover, the decrease in NAA has consistently been associated with outcomes following moderate to severe TBI with several pediatric and adult studies showing a correlation between reduced NAA and long‐term neurological and/or neuropsychological outcome (Ashwal et al., 2000; Babikian et al., 2006, 2018; Dennis et al., 2018; Friedman, Brooks, Jung, Hart, & Yeo, 1998; Garnett et al., 2000; Govind et al., 2010; Holshouser et al., 2019; Kirov et al., 2013; Maudsley et al., 2015, 2017). While changes in NAA are the most consistent MR finding, changes in glutamate (Glu) and gamma‐aminobutyrate (GABA) neurotransmitter systems have also been described. For example, increasing levels of Glx (combined glutamate‐glutamine) are also associated with poor neurological outcome in moderate–severe pediatric and adult TBI (Ashwal et al., 2004; Garnett et al., 2001; Gasparovic et al., 2009; Shutter, Tong, & Holshouser, 2004). There is mounting evidence that MRS provides unique biomarkers of injury, which improves injury detection and prognosis and may be more sensitive following milder injury than imaging alone. Studies of concussion, particularly sports‐related (Alosco et al., 2017), have not only shown the hallmark changes in NAA (Vagnozzi et al., 2010) in the acute stages but also long‐term chronic changes in repetitive head injury (Alosco et al., 2017). In the sports concussion population, reduced Glu levels have also been described acutely in the motor and dorsolateral prefrontal cortex (Henry et al., 2011; Henry, Tremblay, Boulanger, Ellemberg, & Lassonde, 2010). In contrast, there are conflicting findings related to GABA. Friedman et al. (2017) reported increased levels in the frontal lobe but decreased in the posterior cingulate in children with sports‐related concussion, whereas studies in collegiate athletes mid‐season did not find changes in GABA (Lefebvre et al., 2018; Tremblay et al., 2014). Studies have shown that MRS (NAA, Glu, and GABA) is sensitive to sub‐concussive repetitive brain impacts, detecting reductions in Glx and Glu neurotransmitters systems (Bari et al., 2019; Koerte et al., 2015; Lin et al., 2015; Poole et al., 2014). Another method is to use chemical exchange saturation transfer (CEST) imaging which relies on the exchange between mobile protons in amide, amine, hydroxyl groups and bulk water (Dou et al., 2019), generating endogenous or exogenous contrast to measure TBI induced alterations in glutamate and glucose metabolism. While still sparsely used in the field, several preliminary investigations have been performed showing associations with outcome (Ellingson et al., 2019; Mao et al., 2019) with the advantage of providing greater spatial resolution.

#### 3.6.3 Structure–function coupling

In addition to advances in single‐modality studies, multimodal MRI studies combining structural and functional MRI will allow researchers to test directly how structural connectivity (SC) changes in TBI are associated with the alteration of functional connectivity (FC) metrics, allowing more sensitive detection of subtle brain alterations than a single MRI modality. A growing number of studies have integrated SC with FC and quantified the association between SC and FC. This integrated approach is termed structural–functional coupling (SC–FC) (Greicius, Supekar, Menon, & Dougherty, 2008; Honey et al., 2009). The report by Honey et al. (2009) was one of the first studies that applied this quantitative approach, and their empirical results revealed positive correlations (with individual SC–FC correlations ranging from *r* = .39 to .48) between resting‐state functional connectivity and structural connectivity (using diffusion spectrum imaging tractography). This positive SC–FC coupling has also been demonstrated in a recent systematic review on multimodal MRI studies in mammalian brains (Straathof, Sinke, Dijkhuizen, & Otte, 2019), indicating that structural connectivity provides the foundation for the functional system.

To date, only a few multimodal MRI studies have examined the SC–FC relationship in TBI patients, albeit adult brain‐injured populations (e.g., (Caeyenberghs, Leemans, Leunissen, Michiels, & Swinnen, 2013; Costanzo et al., 2014; Gordon et al., 2018; Sharp et al., 2011). For example, Sharp et al. (2011) observed a significant negative correlation between white matter organization of the splenium of the corpus callosum, measured by mean diffusivity, and functional connectivity of the posterior cingulate cortex in chronic TBI patients (Sharp et al., 2011). In other words, TBI patients with more disrupted white matter showed less functional connectivity within the default mode network. In another study, Costanzo et al. (2014) reported that fractional anisotropy of the cingulum was positively correlated with resting‐state fMRI functional connectivity between the left medial frontal cortex and left precuneus/posterior cingulate cortex in patients with combat‐related mild TBI (Costanzo et al., 2014). These findings suggest that changes in functional brain connectivity are directly related to a disrupted neurobiological substrate in TBI patients. However, in these studies, coupling was often quantified by simple Pearson's correlation coefficients between the two MRI modalities. Moreover, this coupling was evaluated at the regional or subnetwork level. Future studies are sorely needed to explore the coupling between structural and functional brain networks in children with TBI. We suggest that advances in MRI data acquisition (e.g., multiband), MRI sequences (e.g., high‐angular resolution diffusion MRI), and analytical approaches (e.g., multilayer network analyses) will lead to an expanded investigation of SC–FC coupling used as a biomarker in TBI than biomarkers based on single neuroimaging modalities.

### 3.7 Incorporating fluid biomarkers

Rapid technical advances have enabled reliable and affordable measurement of blood‐based biomarker panels. The availability of these objective, sensitive, and noninvasive tools to study TBI has the potential to markedly improve diagnosis and patient characterization, enhance our understanding of underlying injury pathophysiology and identify novel therapeutic targets, ultimately, opening up new avenues to the delivery of personalized medicine for TBI (Maas et al., 2017; Mondello & Hayes, 2015).

Over the past decade, a large number of candidate molecular biomarkers that capture multiple pathogenetic pathways underlying complex TBI heterogeneity have been explored and contributed significantly to our overall TBI fund of knowledge and scientific literature (Mondello et al., 2018). Neuronal and glial cell‐derived biomarkers of structural damage or loss have been examined in large prospective and longitudinal studies (Bazarian et al., 2018; Mondello et al., 2014; Undén & Romner, 2010). Measures of glial fibrillary acidic protein (GFAP), S100B, and Ubiquitin C‐terminal hydrolase‐L1 (UCH‐L1), have been shown to differentiate, with high sensitivity and specificity, patients with TBI from healthy and orthopedic comparison groups, as well as identify those with an associated traumatic intracerebral injury (Bazarian et al., 2018; Biberthaler et al., 2006; Diaz‐Arrastia et al., 2014; Papa et al., 2012, 2012; Undén & Romner, 2010; Yue et al., 2019). Importantly, this work supports the concept that a negative biomarker test in patients with recent head injury argues strongly against an associated TBI diagnosis, but a positive result supports a TBI diagnosis even in patients with negative CT scans, in whom more advanced neuroimaging may be indicated to detect subtle brain damage (Gill et al., 2018; Kou et al., 2013; Yue et al., 2019). Beyond their use in the acute diagnosis, GFAP, S100B, UCH‐L1, and neuron‐specific enolase [NSE]) have also proven to be informative in TBI outcome predictions at 6–12 months after moderate to severe TBI (Czeiter et al., 2012; Mercier et al., 2013, 2016; Mondello et al., 2011; Shemilt et al., 2019). However, their prognostic accuracy in patients with mild TBI remains controversial.

In the past few years, the development of ultrasensitive digital immunoassay technologies has enabled the accurate measurement in blood of newly emerging biomarkers of neuroaxonal injury—neurofilament light protein (NF‐L) and tau—that have received considerable interest and shown great potential in sports‐related concussion and mild TBI. Acute assessment (within the first 6 hr) of plasma tau protein after sports concussion identified athletes most likely to experience a prolonged recovery with increased return‐to‐play time (Gill, Merchant‐Borna, Jeromin, Livingston, & Bazarian, 2017; Shahim et al., 2014, 2016). Studies exploring circulating levels of NF‐L after concussive/subconcussive head impacts have demonstrated excellent sensitivity and specificity in discriminating concussed athletes, predicting which individuals will suffer persistent postconcussion symptoms. Further, the biomarker levels correlated with the severity of axonal injury (Shahim, Tegner, Marklund, Blennow, & Zetterberg, 2018; Shahim, Zetterberg, Tegner, & Blennow, 2017). Notably, recent work has identified sex‐dependent biomarker profiles following sports‐concussion (Asken et al., 2018; Gill et al., 2017). Such sex‐related biosignatures appear to reflect distinct biological and pathophysiological mechanisms and ensuing brain vulnerability and, therefore, highlights the urgent need to develop sex‐tailored intervention strategies.

Blood‐based biomarker tests are also increasingly being used in TBI clinical trials (Frankel et al., 2019; Hellewell et al., 2018) and drug development (Kochanek et al., 2016; Mondello et al., 2016) because of their ability to identify underlying injury pathophysiology, and to differentiate distinct subtypes of injury and characterize patient phenotypes (Mondello et al., 2012). In this area of research, they have been proposed as tools to enrich or stratify patient groups (e.g., as predictive biomarkers) in clinical trials, to demonstrate target engagement while revealing evidence of disease modification (surrogate clinical endpoints or pharmacodynamic biomarkers), and to monitor progression and recovery after injury (Browning et al., 2016; Mondello et al., 2016). Mechanistic biomarkers (i.e., biomarkers related directly to injury pathogenesis) such as exosomes (Kenney et al., 2018; Mondello et al., 2018) appear uniquely poised to inform the development of mechanism‐based therapies, and support and implement novel rationally designed clinical trials. While promising, this avenue of precision medicine research is currently in its infancy, and the participation of and collaboration among all stakeholders accompanied by changes in research investment and regulation is required.

Overall, despite striking results and the recent clearance of GFAP and UCH‐L1 by FDA for diagnosis of acute TBI, the road to the widespread, routine clinical use of TBI biomarkers has not been straightforward. Scandinavian countries have been forerunners in the integration of TBI biomarkers (S100B) in clinical guidelines (Unden, Ingebrigtsen, & Romner, 2013). However, in the rest of the world, biomarker–test adoption into clinical practice has lagged far behind because of the perceived insufficient demonstration of improvements in patient management. Streamlining validation of the clinical usefulness, gauging technical advances, the regulatory milieu, and commercial opportunities, and developing new clinical algorithms integrating multiple biomarkers and imaging parameters are the logical and crucial next steps that will lead to the successful broad acceptance and adoption of blood‐based TBI biomarkers in medical practice.

## 4 PRELIMINARY RESULTS FROM ENIGMA BRAIN INJURY

The Military‐Relevant Brain Injury, Pediatric msTBI, and Sports‐Related Head Injury groups each have preliminary results that we briefly report/summarize here. The other subgroups—Intimate Partner Violence, Adult msTBI, Acute Mild TBI, MRS, Resting‐State fMRI, Arterial Spin Labeling, and Cognitive Endpoints group are collecting and collating data, and in the case of the methods groups, proposing and testing methods for combining data across cohorts.

### 4.1 Military Brain Injury

The Military‐Relevant Brain Injury group includes data from 10 cohorts for a total of 803 participants reporting a history of TBI and 609 reporting no history of TBI (8% female). Two of these cohorts are focused on Vietnam War Veterans, while the rest are focused on Active Duty Service Members and Veterans (ADSMV) of more recent conflicts in Iraq and Afghanistan. When comparing all participants reporting any TBI to those reporting none, we found no significant differences. When comparing those reporting a deployment‐related TBI to those reporting no TBI, we found *higher* FA in the TBI group (Dennis, 2019a; Dennis et al., 2018). This result is counterintuitive, as injury is generally associated with lower FA, though some prior studies of mild injury have similarly reported higher FA (Borich, Makan, Boyd, & Virji‐Babul, 2013; Mayer et al., 2012). However, animal data shows higher FA during fiber reorganization after injury, so it is possible that this indicates recovery processes (Jiang, Zhang, & Chopp, 2010; van der Zijden, van der Toorn, van der Marel, & Dijkhuizen, 2008). It is also possible that higher FA is due to uncharacterized pathology such as gliosis. Additional cohorts are being prepared for inclusion, and future work will also include outcome metrics to understand the implications of these results. We also found an interaction between group and sex, with lower FA for female TBI participants.

### 4.2 Pediatric msTBI

Currently, the Pediatric msTBI group includes data from six cohorts, four of which are longitudinal. With data binned into three postinjury windows (acute: <7 weeks, postacute: 2–6 months, chronic: >12 months) there are 44 TBI and 46 control participants in the acute phase, 59 TBI and 86 control participants in the postacute phase, and 102 TBI and 116 control participants in the chronic phase. As in the Military Brain Injury group, preliminary analyses have focused on dMRI. Across all three postinjury windows, lower FA has been found, particularly in central white matter regions like the corpus callosum and corona radiata. Controlling for age at scan, we have also found a significant effect of age at injury in the acute and postacute phases, with higher FA in older patients. We have also found evidence suggesting a group‐by‐sex interaction, with lower FA in female TBI participants (Dennis, 2019b).

### 4.3 Sports‐Related Head Injury

The Sports‐Related Head Injury group currently has data from five cohorts. Of these, four focused on repetitive head hits (RHH) and one on concussion, with a total of 85 RHH versus 27 control and 77 concussed versus 43 control. Three of the RHH studies used a longitudinal design, with scans preseason and postseason. The sports included across these were American football, soccer, hockey, mixed martial arts (MMA), basketball, volleyball, rowing. Preliminary dMRI analyses have not yielded any significant results. Additional datasets are being prepared for inclusion, and future studies will be able to examine concussion as well as RHH.

## 5 FUTURE DIRECTIONS

### 5.1 Harmonization

The broad aim of the ENIGMA Brain Injury group is the meta‐ and mega‐analysis of neuroimaging, neuropsychological, and genetic data across collection sites when available. However, with the development of new subgroups within the Brain Injury Group, our mission is to harmonize both the measures collected and analysis pipelines used across sites. Recently, a number of sub‐groups within our Brain Injury group have provided recommendations for specific neuroimaging and neuropsychological measures sensitive to domains impacted by different mechanisms of brain injury (e.g., head trauma associated with intimate partner violence, concussive and subconcussive impacts associated with sports‐related concussion, blast injury in military personnel and veterans), age of injury (e.g., pediatric, adult), severity of injury (mild, moderate, and severe TBI), or imaging modality (MRS; Bartnik‐Olson et al., in press; Dennis, et al., in press; Esopenko et al., 2019; Koerte et al., 2020; Olsen et al., 2020; Tate et al., 2019). We also provide recommendations on specific genetic variations that warrant investigation across injury populations. The recently formed Cognitive Endpoints group is focused on aggregating data from studies that may have used variable cognitive, clinical, and other outcome measures in their assessment of the various cohorts. In many ways, aggregating this type of data is as critical and challenging as harmonizing the imaging data. There are several methods the Cognitive Endpoints group intends to test (Silverberg et al., 2017). These include (a) limiting analyses to only those sites that collected the same cognitive scale (i.e., Trail Making Test), (b) converting scores within the same domain from different scales to *Z*‐scores based on normative data and calculating an Overall Test Battery Mean, (c) looking at the number and severity of low scores for individual tests within a battery of tests, or (d) calculate a cognitive composite score created by assigning weighted scores based on the degree to which an individual test score deviates from normative expectations. The last two methods could be expected to improve sensitivity to more subtle and often heterogeneous outcomes in mTBI patient groups. By identifying the best methods for aggregating disparate cognitive and clinical measures across studies, we will have increased statistical power in systematically examining the acute, chronic, and long‐term outcomes of brain injury, as well as the influence of genetic variation on injury outcome in patients from multiple populations.

### 5.2 Big data approaches

There is no shortage of factors that could influence outcome, including socio‐demographic variables like sex or socioeconomic status, clinical variables like severity or loss of consciousness, or premorbid considerations like obesity or prior psychiatric disorders, among others. These variables are too numerous to examine in simple group *t* tests or regressions, and, likely, the associations between each of these and “outcome” are not clear cut. Machine learning methods like support vector machines (SVMs) or deep learning allow for the identification of patterns across large datasets. While there have been some neuroimaging publications identifying individual factors that are associated with outcome (Dennis et al., 2017; Ewing‐Cobbs et al., 2016; Prasad, Ewing‐Cobbs, Swank, & Kramer, 2002; Yuh et al., 2013), a comprehensive, data‐driven approach has been beyond the capacity of studies to date. There have been several implementations of machine learning approaches within the ENIGMA Consortium already. The ENIGMA BrainAGE group has built an algorithm to predict chronological age from morphometric data. The ENIGMA MDD group found that the brains of depressed individuals appeared nearly a year older than controls (Han et al., 2020). Applying this to TBI will yield a generalized measure of the impact of TBI on brain structure. The ENIGMA Bipolar, Addictions/Substance Use, ADHD, and 22q11.2 Deletion Syndrome groups have all used SVMs to classify cases versus controls from structural data (Mackey et al., 2019; Nunes et al., 2018; Sun et al., 2018; Zhang‐James et al., 2019). Another approach that may be particularly useful for TBI given the heterogeneity and our aim to identify clinically meaningful patient subtypes is symmetric multivariate linear reduction (SyMILR, https://github.com/ANTsX/ANTsR/blob/master/R/multiscaleSVDxpts.R#L2743). SyMILR aims to reduce high dimensional, multimodal data and can take in multiple types of inputs. Through principal components analysis (PCA) decomposition, MR images can be reduced to components that reflect the variability across the image. With SCCAN (sparse canonical correlation analysis for neuroimaging), this decomposition can be run across several inputs (e.g., T1‐weighted image and T2‐weighted image, or FA image and neurocognitive testing), reducing them in a way that maximizes the correlation between the resulting components. The result is a set of components for each input that captures the variability in the data and could point to patterns of disruption that bridge modalities.

### 5.3 Cross‐disorder analyses

One benefit of being part of the broader ENIGMA consortium is the potential for cross‐disorder analyses. There are a number of psychiatric and developmental disorders that may be premorbid vulnerabilities for sustaining a TBI, such as attention‐deficit/hyperactivity disorder (ADHD) or substance use disorders, and others that are frequently comorbid with TBI or may develop after an injury, such as posttraumatic stress disorder (PTSD) and major depressive disorder (MDD). The ENIGMA consortium has established working groups for each of these disorders (Dennis et al., 2019; Hoogman et al., 2017; Logue et al., 2017; Mackey et al., 2019; Schmaal et al., 2016; van Velzen et al., 2019). Formal cross‐disorder analyses have begun in the ENIGMA consortium with working groups focused on schizophrenia and bipolar disorder. These disorders have partially overlapping symptoms and a strong genetic correlation (de Zwarte et al., 2019). In analysis including participants with schizophrenia or bipolar disorder, first‐degree relatives of individuals with schizophrenia or bipolar disorder, and healthy controls, they found different patterns of structural alterations in the relatives of individuals with schizophrenia or bipolar disorder. These results indicate that, despite overlap in symptoms and genetic basis, schizophrenia and bipolar disorder do not appear to stem from similar neurodevelopmental trajectories. The PGC‐PTSD group (the neuroimaging working group formed the basis of the PGC‐ENIGMA PTSD working group) has established a TBI working group as well, focused on the genetic overlap between PTSD and outcome after TBI. There is significant overlap in cohorts between the PGC‐ENIGMA‐PTSD working group and the ENIGMA Military Brain Injury group, facilitating cross‐disorder analyses.

## 6 CONCLUSIONS

TBI is a major public health issue worldwide, and while there have been many excellent studies using neuroimaging to advance our understanding of injury and recovery processes and factors that affect them, there remains a large amount of unexplained variance in outcome. This is due in part to small sample sizes, as neuroimaging data is expensive to collect, and some populations (e.g., pediatric patients) are harder to recruit in large numbers. Through the collaborative framework of ENIGMA, however, researchers from around the world can work together to address open questions in the field, processing data in a harmonized manner and pooling intellectual resources to optimize our approach. While there are a number of clinical questions that may only be addressed through in‐depth phenotyping in smaller studies, the ENIGMA Brain Injury working group represents a significant increase in statistical power to answer other questions such as sex differences, comorbidities, and the existence of patient subtypes. We will also advance the field through the development of new processing pipelines tailored to the injured brain and provide guidance and recommendations for data collection going forward. The ENIGMA Brain Injury working group is open to all interested in participating, and we invite researchers to contact the authors for details on how to be involved.

## CONFLICT OF INTEREST

P. M. T. received partial research support from Biogen, Inc. for research unrelated to this manuscript. The views expressed in this manuscript are those of the authors and do not reflect the official policy of the Department of Army/Navy/Air Force, Department of Defense, or U.S. Government.

## Data Availability

Data sharing is not applicable to this article as no new data were created or analyzed in this study.
